# Does income inequality moderate the effect of fintech development on renewable energy consumption?

**DOI:** 10.1371/journal.pone.0293033

**Published:** 2023-11-27

**Authors:** Duc Hong Vo, Anh Tuan Pham, Thao Tran, Nam Thanh Vu

**Affiliations:** 1 Research Centre in Business, Economics & Resources, Ho Chi Minh City Open University, Ho Chi Minh City, Vietnam; 2 International School of Business, University of Economics Ho Chi Minh City, Ho Chi Minh City, Vietnam; The University of Hong Kong, HONG KONG

## Abstract

Fintech development is generally considered as an effective mechanism to promote the consumption of renewable energy sources. The relationship between fintech development and renewable energy consumption have been examined in previous studies. However, the moderating effect of income inequality on this relationship has largely been ignored in the existing literature. As such, this study is conducted to shed light on this moderating effect. Two estimation techniques, including the two-step system generalized method of moments (GMM) and the method of moments quantile regression (MMQR), were used on a sample of 65 countries from 2013 to 2019. Our findings reveal that fintech development plays a vital role in promoting the consumption of renewable energy sources. However, it is crucial to recognize that rising income inequality may hinder the potential positive effects of fintech development on renewable energy consumption. A threshold of income inequality should be maintained to ensure that the positive effect of fintech development on increased renewable energy consumption is not compromised. Policy implications have emerged based on the findings from this study regarding promoting fintech development towards green economic growth and sustainable development.

## 1. Introduction

**Fig 1:**
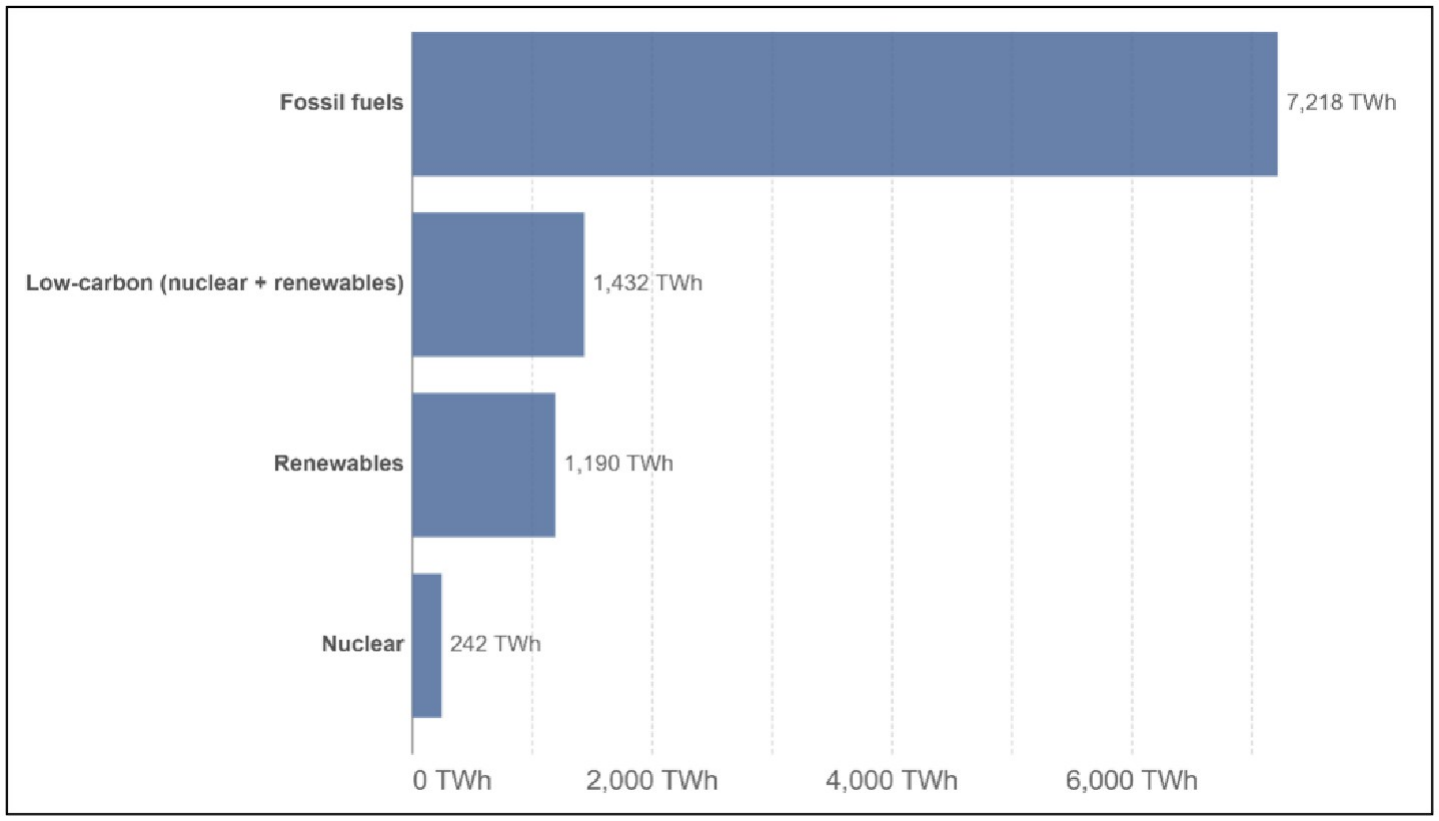
World primary energy consumption by sources in 2021. *Source*: Our World in Data [5].

Climate change is currently one of humanity’s most vexing developmental challenges [1]. It is driven by greenhouse gases emitted from human activities. Anthropogenic pollution emissions have shown an upward trend over several decades [2]. This increase has exerted substantial pressure on the environment, and if the trend persists without any signs of abatement, humans will suffer the consequences of climate change, such as extreme temperatures or frequent natural disasters. As such, efforts are being made to find feasible solutions to address these environmental issues. One of the solutions is to shift energy consumption away from non-renewable energy sources emitting substantial greenhouse gases and instead focus on renewable energy sources emitting little to no polluting emissions [3, 4]. Burning fossil fuels, including coal, oil, and gas, is the primary source of carbon dioxide emissions. However, the global energy system relies heavily on fossil fuels [5]. Global energy consumption continues to grow, with more than 80 per cent coming from fossil fuels, and just less than one-fifth of it is from renewable sources (Fig 1).

The fourth industrial revolution has accelerated the technological innovation progress, from which the financial sector is considered to benefit substantially [6]. This progress has led to rapid fintech development. Various benefits of fintech development have been documented [7–11]. However, fintech development still has some drawbacks. For instance, Bao and Huang found that fintech platforms can customize messages for different borrowers to encourage timely repayment [12]. However, they also highlight that the delinquency rate of fintech loans may increase significantly during times of crisis, while the rate for traditional loans remains unchanged [13]. In this regard, research is devoted to investigating the relationship between fintech and energy consumption [14]. The emergence of cryptocurrencies fostered by fintech development has increased the demand for electricity consumption [15]. As a result, the surge in polluting emissions, coupled with the growing energy demand, has led to the growing trend of global surface temperatures, possibly serving as the primary driver of climate change. On the other hand, fintech development can also promote using renewable energy sources. Specifically, it influences the consumption decisions of individuals and investment opportunities in the renewable energy sector [9]. Therefore, fintech development plays a crucial role in improving environmental quality by enabling the widespread adoption of renewable energy sources and the proliferation of low-carbon projects. Income inequality also contributes significantly to the adoption of renewable energy sources. This is because income inequality is a crucial determinant of renewable energy consumption and is closely linked to sustainable development policies. Existing literature shows two complementary channels through which income inequality can influence the environment: the economic and political channels [16, 17]. Income inequality influences renewable energy consumption through the economic channel by shaping social norms such as individualism, consumerism, and short-termism. On the other hand, income inequality can also impact renewable energy consumption through various political factors, including institutional systems and elite groups in society.

The literature review indicates that both fintech development and income inequality play crucial roles in promoting the adoption of renewable energy and improving environmental quality. Various attempts have been made to analyse either the role of fintech development or the effect of income inequality on environmental quality proxied by carbon dioxide emissions or renewable energy consumption. However, the combined effect of fintech development and income inequality on renewable energy consumption has largely been neglected in the existing literature. As such, this study is conducted to examine this combined effect to provide additional evidence to the literature on this important topic.

Particularly, our study sheds light on the role of income inequality in the relationship between fintech development and renewable energy consumption. The method of moments quantile regression (MMQR) is the main estimation method in this analysis. This MMQR method allows the observation of heterogenous effects of fintech and income inequality across different levels of renewable energy consumption. Using a sample of 65 countries from 2013 to 2019, findings from our analysis reveal that fintech development can promote the adoption of renewable energy sources. However, rising income inequality may counteract the important effect of fintech development on renewable energy consumption. These findings highlight the role of income inequality in moderating the effect of fintech development on renewable energy consumption. Moreover, findings from this study imply the critical thresholds for income inequality that should not be exceeded to ensure that the positive impact of fintech development on renewable energy consumption can be realized. Our findings have largely remained unchanged across additional robustness analyses. These findings can provide valuable guidance for countries attempting to achieve green growth and sustainable development.

Our contributions to the existing literature are threefold. *First*, we address the gap in environmental and public policy research by shedding light on the combined effects of fintech development and income inequality on the environment, which have received limited attention thus far. The existing literature has documented limited insights into this effect. However, previous studies have primarily focused on analysing the individual effects of fintech development or income inequality on renewable energy consumption without considering their dynamic interaction. *Second*, we provide a fresh perspective on the interrelationship between fintech development, income inequality, and the environment using renewable energy consumption–a positive indicator of environmental sustainability, rather than relying on carbon dioxide emissions–a negative indicator which has been widely used in previous studies. *Third*, we employ a novel estimation technique called the method of moments quantile regression to examine this interrelationship. This technique enables the observation of heterogeneous effects across various conditional quantile distributions. Policy implications have emerged based on the findings of our analysis regarding the link between innovative financial services and the adoption of renewable energy sources, particularly in the context of rising income inequality. These policy implications carry a significant value for countries with substantial pollutant emissions as they strive to transition towards a low-carbon economy while pursuing sustainable economic growth and development.

The structure of this paper is as follows. Following this introduction, section 2 discusses the literature review. Section 3 presents the research methodology of the study. The empirical analysis and robustness check are respectively provided in sections 4 and 5, followed by the conclusions in section 5 of the paper.

## 2. Literature review

This section will review the existing empirical literature on the interrelationship between fintech, income inequality, and energy usage. Although a limited number of studies have examined this specific interrelationship, the existing literature provides valuable insights into the potential dynamics and implications of this interaction.

In the emergent literature, much research is devoted to investigating the relationship either between fintech and the environment or between fintech and energy consumption [14]. Findings from various studies show that fintech development has a negative effect on the environment [15, 18, 19]. They argue that the emergence of the cryptocurrency market due to fintech development has fostered the demand for electricity and energy consumption. Consequently, polluting emissions have skyrocketed, leading to the rise in global surface temperatures, which is the primary cause of climate change. For instance, Erdogan et al. [15] examine the effects of Ethereum and Ripple on the environment and find that there are pollution-enhancer asymmetric effects from cryptocurrency demand to environmental degradation. Likewise, Okorie [19] investigates the effects of Bitcoin and Ethereum on electricity demand in the leading crypto-mining economies, including the United States, China, and Japan. Findings reveal that the development of cryptocurrency markets directly leads to higher energy usage and carbon emissions. Similarly, Baur and Oll [20] argue that although carbon intensity measure is common in financial discipline, it remains scant in the bitcoin literature. As such, they argue for a bitcoin’s absolute emissions in mitigating climate change. Their findings reveal that the carbon intensity from bitcoin investments can be less than those of traditional equity investments, which diminishes the portfolio’s total carbon footprint.

Bolstering fintech development, in contrast, can promote renewable energy usage because it influences the consumption decisions of individuals and investment opportunities in the renewable energy sector [9, 21, 22]. For instance, innovative financial technology offers new ways for citizens to get involved in green energy projects, such as crowdfunding for financing or cryptocurrency as alternative means of payment. Croutzet and Dabbous [9] attempt to quantify the impact of fintech development on renewable energy consumption and confirm the positive effect. That is, fintech development has the potential to serve as a solution to enhance the integration of renewable energy sources within the energy mix. Teng and Shen [21] similarly investigate the effect of fintech on energy efficiency and show that fintech development indirectly promotes energy efficiency through increased renewable energy consumption. Likewise, Tao et al. [14] seek to explore whether fintech development is beneficial for the environment. These authors confirm that fintech development can reduce greenhouse gas emissions and facilitate the transition towards a low-carbon economy.

Meanwhile, considering the effect of income inequality when promoting renewable energy consumption is also of the utmost importance. Income inequality is an important factor contributing to the use of renewable energy and is closely related to sustainable development policies. The existing literature shows two complementary channels through which income inequality can affect the environment. These channels include economic and political channels [16, 17]. On the one hand, through the economic channel, income inequality affects renewable energy consumption by affecting social norms (i.e., individualism, consumerism, and short-termism). Societies characterized by high-income inequality tend to lack collective actions and lean towards individualism, resulting in the absence of environmental sensitivity and leading to rent-seeking behaviour and intensive use of non-renewable energy. Accordingly, people in such societies do not have incentives to pursue renewable energy sources as they concern more about short-term benefits instead of the long-term consequences of their consumption activities [23]. On the other hand, income inequality can also affect renewable energy consumption through the political channel. This effect can be negative or positive. Income inequality can signify a society with weak institutional systems without incentives to pursue efficient investments or effective environmental policies. Particularly, inefficient investments can reflect a limited investment in alternative energy sources, affecting the adoption of renewable or clean energy sources. One possibility is that due to ineffective environmental policies, elite groups in such societies tend to involve more in activities that benefit them but harm the environment [24]. Consequently, the use of renewable energy can be inhibited. An alternative possibility, in contrast, is that elite groups in such society will be more concerned about their environment, thereby influencing the government to tighten environmental regulations [25]. As a result, the use of renewable energy can be promoted.

Our literature review indicates that the existing literature has focused on the potential impact of fintech development and income inequality on renewable energy consumption, albeit with limited empirical evidence. Previous studies have provided insights into the separate impacts of fintech development and income inequality. However, there is a gap in the existing literature regarding a comprehensive examination of the combined effect of fintech development and income inequality on renewable energy consumption and the underlying mechanisms for the impact.

## 3. Methodology

### 3.1. Model

This study examines the individual effects of fintech development, income inequality, and their combined effect on renewable energy consumption for 65 countries from 2013 to 2019. Our empirical model is developed considering relevant previous studies [17, 26, 27] as follows.

$$\text{RE}{\text{C}}_{\text{i},\text{t}}=\alpha_{\text{i}}+\beta_{1}\text{FI}{\text{N}}_{\text{i},\text{t}}+\beta_{2}\text{GIN}{\text{I}}_{\text{i},\text{t}}+\beta_{3}\text{FIN}*\text{GIN}{\text{I}}_{\text{i},\text{t}}+\beta_{4}\text{PCGD}{\text{P}}_{\text{i},\text{t}}+\beta_{5}\text{FD}{\text{I}}_{\text{i},\text{t}}+\beta_{6}\text{PO}{\text{L}}_{\text{i},\text{t}}+\beta_{7}\text{C}{\text{O}}_{2\text{i},\text{t}}+\varepsilon_{\text{i},\text{t}}$$

where i = 1, 2, 3, … N for each country and t = 1, 2, 3, … T for each year in the panel. REC_i,t_ is the dependent variable, representing renewable energy consumption for country i in year t. The main independent variables of interest include fintech development (FIN_i,t_) and income inequality (GINI_i,t_) for country i in year t. The interaction term FIN*GINI_i,t_ is included to observe whether the effect of fintech development on renewable energy consumption depends on income inequality. We also consider other important determinants of renewable energy consumption, including: (i) economic factors proxied by per capita income (PCGDP_i,t_) and foreign investment (FDI_i,t_); (ii) political factors captured by political constraints (POL_i,t_); and (iii) environmental factor represented by carbon dioxide emissions (CO_2i,t_).

### 3.2. Data

We collect data from the following sources: the World Development Indicators (WDI) from the World Bank’s database; the Wharton Research Data Services (WRDS), the Standardized World Income Inequality Database (SWIID) of Harvard Dataverse [28]; and the Bank for International Settlements (BIS) [29]. Table 1 presents the list of selected countries. Tables 2 and 3 present the descriptions and the descriptive statistics of variables used in our analysis.

**Table 1:** List of countries.

High-income countries		Middle- and low-income countries	
Argentina	Latvia	Brazil	Philippines
Australia	Lithuania	Bulgaria	Poland
Austria	Netherlands	Colombia	Russia
Belgium	New Zealand	Egypt	South Africa
Canada	Norway	El Salvador	Thailand
Chile	Panama	Georgia	Turkey
Czechia	Portugal	Ghana	Zambia
Denmark	Singapore	India	Zimbabwe
Estonia	Slovakia	Indonesia	Burkina Faso
Finland	Slovenia	Jordan	Malawi
France	Spain	Kenya	Mali
Germany	Sweden	Malaysia	Mozambique
Ireland	Switzerland	Mexico	Rwanda
Israel	United Arab Emirates	Mongolia	Tanzania
Italy	United Kingdom	Myanmar	
Japan	United States	Paraguay	
Korea	Uruguay	Peru	

**Table 2:** The descriptions of variables.

Variable		Description	Source
**Dependent variable**			
Renewable energy consumption	REC	Renewable energy consumption (% of total final energy consumption)	WDI
**Independent variables**			
Income inequality	GINI	Gini coefficient of the disposable income	SWIID
Fintech development	FIN	Natural logarithm of FinTech credit (Million US$)	BIS
Per capita income	GDP	GDP per capita (constant 2015 million US$)	WDI
Foreign direct investment	FDI	Foreign direct investment, net inflows (% of GDP)	WDI
Political constraints	POL	Political constraint index (POLCONV)	WRDS
Carbon dioxide emissions	CO_2_	CO_2_ emissions (metric tons per capita)	WDI

**Table 3:** Descriptive statistics.

Variable	Obs	Mean	Std. Dev.	Min	Max
REC	455	30.095	25.128	0.1	88.47
FIN	418	3.074	2.466	0	11.159
GINI	437	36.614	8.078	22.6	62.7
GDP	455	21.658	21.288	0.366	87.124
FDI	455	4.008	7.907	-37.173	81.248
POL	455	0.594	0.306	0	0.891
CO2	455	5.338	4.43	0.051	22.357

Our sampling approach produces an unbalanced panel for 65 countries worldwide from 2013 to 2019. Specifically, our dataset has missing values on two variables, namely fintech development (FIN_i,t_) and income inequality (GINI_i,t_). We consider the use of an unbalanced panel since missing observations are common features in quantitative analysis [30]. In this study, our empirical analysis is conducted using Stata statistical software, which is known for its ability to handle unbalanced datasets. This software employs listwise deletion, which excludes missing observations on any variables, to facilitate the analysis as a whole case.

Our dependent variable is renewable energy consumption, proxied by renewable energy consumption as a proportion of total final energy consumption. This measure is widely used in the existing literature on renewable energy consumption [31–33]. The main independent variables in the study include fintech development and income inequality. Fintech development is measured by the natural logarithm of the total volume of fintech credit in US$ million [11, 29, 34]. The core business of fintech credit is from the financial services, and the utilized fintech credit covers all loan-based business models, including (i) peer-to-peer (P2P) lending, (ii) balance sheet lending, (iii) invoice trading, (iv) debt-based securities, and (v) mini-bonds. Meanwhile, income inequality is measured by the most popular proxy in the income inequality literature, the Gini coefficient of disposable income, ranging from 0 –perfect equality to 100 –perfect inequality [8, 34, 35]. Other control variables include per capita income, foreign direct investment, political constraints, and carbon dioxide emissions. These variables have been widely used in the literature as the key determinants of renewable energy consumption [17, 27, 32, 36, 37].

### 3.3. Our empirical estimations

Our econometric strategy seeks to (i) examine how fintech development and income inequality affect renewable energy consumption, (ii) investigate whether the effect of fintech development on renewable energy consumption is contingent upon income inequality, (iii) observe the heterogenous effects of fintech development and income inequality across different distributions of renewable energy consumption, and (iv) address potential endogeneity issues in panel data analysis.

We initially use the fixed-effects model (FEM) and the two-step system generalized method of moments (GMM) to address biases from potential endogeneity such as unobserved heterogeneity, omitted variable bias, and measurement error [38, 39]. These estimation methods enable us to provide robust and unbiased estimates of the interrelationship between fintech development, income inequality, and renewable energy consumption.

Later, the method of moments quantile regression (MMQR) is used to examine the heterogeneous and distributional effects across different quantiles [40]. The merit of this MMQR approach is to detect the covariance effects under conditional heterogeneity and allow individual effects to affect the entire distribution. In addition, this technique is generally known to be pertinent to the models anchored by individual effects and endogenous independent variables [41].

## 4. Results

### 4.1. Panel data regression

We initially examine the linkages using panel data estimation techniques that can address potential endogeneity. The results of our estimations are presented in Table 4. The estimated coefficients of fintech development (FIN) and income inequality (GINI) are positive. These coefficients are highly significant, indicating that these factors drive renewable energy consumption significantly. The estimated coefficients of the interaction term (FIN*GINI) are significant and negative, suggesting that income inequality can limit the positive effect of fintech development on renewable energy consumption.

**Table 4:** The effect of fintech development and income inequality on renewable energy consumption using the fixed-effects model (FEM) and the two-step system GMM (GMM).

	FEM		GMM	
REC_t-1_			0.981***	0.979***
			(0.023)	(0.023)
FIN	1.477***	0.826*	1.256***	1.072***
	(0.493)	(0.417)	(0.469)	(0.408)
GINI	0.978**	0.901***	0.109	0.108**
	(0.402)	(0.288)	(0.067)	(0.054)
FIN*GINI	-0.035**	-0.022*	-0.032**	-0.028**
	(0.015)	(0.013)	(0.013)	(0.011)
GDP		0.164***		0.016**
		(0.038)		(0.007)
FDI		0.012		-0.001
		(0.009)		(0.007)
POL		1.400		0.222
		(1.325)		(0.433)
CO2		-1.529***		-0.060
		(0.303)		(0.057)
Constant		-1.035	-3.820*	-3.782**
		(11.046)	(1.970)	(1.671)
Threshold	42.2	**37.9**	39.3	**38.1**
Observations	404	404	356	356
Countries	65	65	65	65
Instruments	--	--	28	32
AR(2) [p-value]	--	--	0.386	0.384
Sargan [p-value]	--	--	0.414	0.518
Hansen [p-value]	--	--	0.170	0.219

Notes. **REC** denotes renewable energy consumption; **FIN** captures fintech development; **GINI** captures income inequality; **GDP** represents per capita income; **FDI** represents foreign direct investment; **POL** represents political constraints; **CO**_**2**_ represents carbon dioxide emissions.

Standard errors are reported in paratheses.

***, **, * denote significant levels at 1 per cent, 5 per cent, and 10 per cent, respectively.

**Fig 2:**
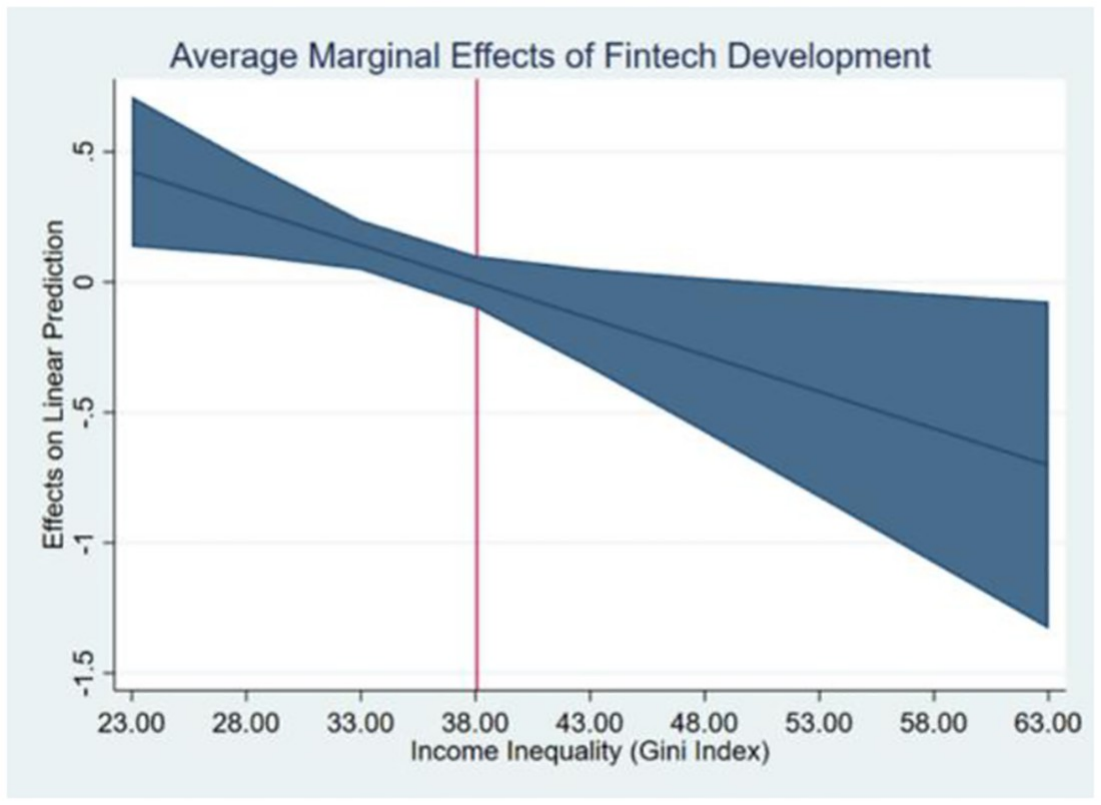
Marginal effects of fintech development at different levels of income inequality.

We now estimate an income inequality threshold that should not be surpassed. Fig 2 presents the average marginal effects of fintech development on renewable energy consumption at different levels of income inequality. It is important to highlight that if the Gini index exceeds the computed threshold of approximately 38 (1.072/0.028), the effect of fintech development on renewable energy consumption will shift from positive to negative, as illustrated in Fig 2.

Our findings align with existing empirical evidence, which considers fintech development an effective means to encourage renewable energy consumption [9, 21]. Fintech development introduces new and innovative financial services, which profoundly affect the decision-making of individuals and businesses and, thus, renewable energy consumption. Particularly, fintech development provides new forms of capital funding and risk management, creating an environment for fintech start-ups towards green projects and giving rise to the demand and supply of renewable energy sources. Individuals can also make small contributions to low-carbon projects and initiatives through innovative fintech platforms, which facilitate the financing and investment for such projects and initiatives.

Income inequality can potentially trigger the adoption of renewable energy through investments from the top income group [16]. However, rising income inequality may counteract the potential benefits of fintech development on renewable energy consumption. This observation aligns with the existing literature, indicating that limited financial resources in countries with high-income inequality hinder the adoption of renewable energy sources [26, 42]. One possible explanation is that income inequality often results in a significant portion of the population, particularly the low-income group, having limited access to fintech platforms and services. Accordingly, investing in green projects and initiatives is more challenging for individuals and businesses. In addition, societies characterized by high-income inequality often have weak institutions and lack collective action. In such circumstances, environmental policies are less effective, citizens are less environmentally conscious, and there are fewer incentives to pursue renewable energy. Consequently, financial resources are prioritized for meeting basic needs rather than investing in renewable energy sources.

### 4.2. Panel quantile regression

In this section, we examine the heterogeneous effects of fintech development and income inequality across the conditional distributions of renewable energy consumption. Table 5 presents the results using panel quantile estimations.

**Table 5:** The effect of fintech development and income inequality on renewable energy consumption using the method of moments quantile regression (MMQR) with country-fixed effects.

	Location	Scale	Q10	Q25	Q50	Q75	Q90
FIN	0.826***	-0.143	1.058***	0.964***	0.829***	0.693***	0.612**
	(0.204)	(0.115)	(0.273)	(0.229)	(0.204)	(0.232)	(0.269)
GINI	0.901***	-0.108	1.075***	1.004***	0.903***	0.801***	0.740***
	(0.155)	(0.087)	(0.207)	(0.175)	(0.155)	(0.177)	(0.205)
FIN*GINI	-0.022***	0.003	-0.027***	-0.025***	-0.022***	-0.019***	-0.017**
	(0.006)	(0.003)	(0.008)	(0.007)	(0.006)	(0.007)	(0.008)
GDP	0.164***	0.009	0.150***	0.156***	0.164***	0.172***	0.177***
	(0.029)	(0.017)	(0.039)	(0.033)	(0.029)	(0.034)	(0.039)
FDI	0.012**	-0.001	0.014*	0.013**	0.012**	0.012*	0.011
	(0.006)	(0.003)	(0.008)	(0.006)	(0.006)	(0.007)	(0.008)
POL	1.400	-0.197	1.718	1.589	1.403	1.216	1.105
	(1.089)	(0.614)	(1.458)	(1.227)	(1.089)	(1.240)	(1.439)
CO2	-1.529***	-0.158	-1.273***	-1.377***	-1.526***	-1.676***	-1.766***
	(0.186)	(0.105)	(0.249)	(0.209)	(0.186)	(0.212)	(0.246)
Constant	-1.035	5.655*	-10.189	-6.471	-1.137	4.227	7.432
	(6.070)	(3.422)	(8.131)	(6.844)	(6.091)	(6.921)	(8.021)
Threshold	37.5	NA	39.2	38.6	37.7	36.5	36
Observations	404	404	404	404	404	404	404

Notes. **REC** denotes renewable energy consumption; **FIN** captures fintech development; **GINI** captures income inequality; **GDP** represents per capita income; **FDI** represents foreign investment; **POL** represents political constraints; **CO2** represents carbon dioxide emissions.

Standard errors are reported in paratheses. Symbols ***, **, * denote significant levels of 1 per cent, 5 per cent, and 10 per cent, respectively.

The findings are consistent with those from the previous analysis. Our empirical results confirm a positive relationship between fintech development (FIN) and renewable energy consumption. This effect remains significant across all quantiles of renewable energy consumption. The estimated coefficient of fintech development is observed to diminish in magnitude from lower to higher quantiles. Furthermore, the interaction term (FIN*GINI) exhibits a negative and highly significant association with renewable energy consumption. Our findings align with existing studies that confirm that innovative financial means can contribute to adopting renewable energy, but developmental issues in society may damper the underlying positive effect [26, 43, 44].

Per capita income (GDP) exhibits a positive and significant relationship with renewable energy consumption. An increase in per capita income is associated with increased energy consumption, including renewable energy sources [45]. Another possible interpretation is that higher income is synonymous with larger financial resources for renewable energy projects and initiatives. Although we only consider the linear term of per capita income, its effect can be nonlinear, which is also known as the environmental Kuznets curve hypothesis [46]. However, whether such a nonlinear relationship exists depends on the country’s characteristics and stage of development. Foreign direct investment (FDI) also has a positive relationship with renewable energy consumption, highlighting the role of international flows in facilitating the transfer of technology and knowledge necessary for adopting renewable energy [47]. Our results also indicate a negative and significant relationship between renewable energy consumption and carbon dioxide emissions (CO_2_). This negative relationship is expected because countries with high polluting emissions tend to address environmental concerns by transitioning to low-carbon energy sources [48].

## 5. Robustness check

In this section, we conduct additional analysis to ensure the robustness of our findings. Two alternative panel techniques are employed: the feasible generalized least squares (FGLS) and the panel corrected standard errors (PCSE). These techniques are used to address heteroskedasticity and serial correlation issues.

Table 6 presents the empirical results of the robustness analysis. We note that findings from our robustness analysis are consistent with our main findings. The individual effect of fintech development and income inequality on renewable energy consumption is positive and significant, whereas their combined effect is negative and significant. The implications of these results remain the same as our previous interpretations of the empirical results.

**Table 6:** The robustness analysis—The effect of fintech development and income inequality on renewable energy consumption using the FGLS and the PCSE.

	FGLS		PCSE	
FIN	2.457***	2.378***	4.091*	4.571**
	(0.829)	(0.638)	(2.317)	(1.862)
GINI	1.104***	0.444***	1.346***	0.738***
	(0.099)	(0.085)	(0.279)	(0.225)
FIN*GINI	-0.122***	-0.094***	-0.182***	-0.161***
	(0.024)	(0.019)	(0.065)	(0.052)
GDP		0.082***		0.111*
		(0.031)		(0.063)
FDI		-0.139*		-0.140
		(0.073)		(0.110)
POL		5.490**		0.153
		(2.308)		(4.140)
CO2		-3.111***		-3.053***
		(0.155)		(0.285)
Constant	-8.253**	25.128***	-13.977	19.723**
	(3.281)	(3.991)	(10.135)	(9.611)
Observations	404	404	404	404

Notes. **REC** denotes renewable energy consumption; **FIN** captures fintech development; **GINI** captures income inequality; **GDP** represents per capita income; **FDI** represents foreign investment; **POL** represents political constraints; **CO2** represents carbon dioxide emissions.

Standard errors are reported in paratheses. Symbols ***, **, * denote significant levels of 1 per cent, 5 per cent, and 10 per cent, respectively.

## 6. Conclusions

The negative consequences of climate change have always been at the forefront of environmental issues and discussions. Climate change has been a global climate policy concern. Countries have endeavoured to encourage the widespread adoption of renewable energy sources. However, renewable energy adoption rates remain relatively low compared to the established targets. As such, it is crucial to identify the key determinants of renewable energy consumption to inform policy decisions. In this study, we contribute to the existing literature by examining the impact of fintech development, conditioning upon income inequality, on renewable energy consumption on a global sample.

We employ dynamic panel and panel quantile estimation techniques to address the potential bias arising from endogeneity issues. Our sample includes 65 countries from 2013 to 2019. Findings from our analysis confirm a positive correlation between fintech development and renewable energy consumption, implying that fintech development supports increased consumption of renewable energy. However, income inequality may diminish this positive effect. In addition, the positive effect of fintech development diminishes from lower to higher quantiles of renewable energy consumption. Nevertheless, no evidence of distributional heterogeneity has been found in our analysis. Furthermore, our study identifies an income inequality threshold that should not be surpassed to maintain the positive effect of fintech development on renewable energy consumption.

Our study highlights that adopting renewable energy sources can be promoted through fintech development. Specifically, fintech development has the potential to reshape the financial sector landscape, influencing the decision-making processes of individuals and businesses and ultimately leading to increased uptake of renewable energy sources. Nonetheless, it is crucial to reduce income inequality to prevent a counterproductive impact on the link between fintech development and renewable energy consumption. High-income inequality can limit the availability of innovative financial resources for individuals and businesses to utilize renewable energy sources. Accordingly, policy implications towards the green economy have emerged based on the findings from this study on the ground of three key determinants: the adoption of low-carbon energy sources, the innovation of financial sectors, and the progress towards alleviating income inequality.

Identifying the determinants of renewable energy consumption is essential, as promoting low-carbon energy sources can contribute to reducing polluting emissions and fighting against climate change. Countries must develop and implement policies that support individuals and businesses in transitioning towards low-carbon or renewable energy sources to facilitate progress. Governments can consider the following actions to facilitate the use of low-carbon energy sources, including implementing a carbon tax, investing in green technologies, providing subsidies and incentives for renewable energy infrastructure, and promoting adopting environmentally friendly technologies. These policies have been generally considered to positively affect environmental quality [27, 49]. Besides, countries can encourage green lifestyles and raise public awareness about environmental degradation with minimal efforts to address rising global surface temperature.

The advancement of financial sectors has resulted in introduction of innovative financial services, also known as fintech. Fintech development is considered a very effective way to facilitate the use of low-carbon energy sources [9, 49]. In this regard, countries must invest in developing and expanding digital technologies to support the financial sector. Accordingly, a wider range of innovative financial services will significantly promote renewable energy consumption, improving environmental quality. Moreover, fintech development offers new capital and risk management tools, streamlines information acquisition and dissemination, and optimizes renewable energy production and adoption. Hence, financial policies should be integrated with technological and environmental policies to effectively assist countries in progressing towards a green economy. Countries can provide appropriate financial incentives for firms to invest in technologies that support green growth and sustainable development. For instance, policies such as tax credits or subsidies for investments and research in green technologies could be considered.

Besides, the progress towards low levels of income inequality is also crucial in achieving green growth and sustainable development. Countries must consider income inequality when utilising fintech development to uptake renewable energy sources. Although income inequality may initially provide a positive effect on renewable energy consumption via investments from the top income groups, these effects are not sustainable in the long run. Instead, income inequality may lower renewable energy adoption. A society characterized by high-income inequality often exhibits weak institutions and a lack of collective actions. In such circumstances, individuals and businesses may have limited financial resources to transition to low-carbon energy sources. As such, countries should pay close attention to the role of income inequality to improve the efficacy of policies promoting fintech development towards a green economy. Governments can address income inequality through various actions, including promoting equal access to education, increasing public spending on active labour market policies, encouraging immigrant integration, and implementing equitable tax systems that support inclusive growth.

Our work, however, exhibits limitations, which provide avenues for further research. Our analysis only considers a single measure of renewable energy consumption, limiting our findings’ scope. Future studies could utilize alternative metrics of renewable energy consumption regarding different types and energy sources. Additionally, it is worth noting that our findings are based on a sample of 65 countries, which may hinder the generalization of the inferences to country-specific contexts. As such, future research can analyse the established relationships within individual countries or groups of countries sharing similar characteristics. Furthermore, further work can investigate how different and significant the varying levels of fintech development impact the adoption of renewable energy. This aspect is particularly important given that our study has examined various levels of renewable energy consumption and income inequality through conditional distribution analysis.
